# A Literature Review of Wheelchair Transportation Safety Relevant to Automated Vehicles

**DOI:** 10.3390/ijerph19031633

**Published:** 2022-01-31

**Authors:** Kathleen D. Klinich, Miriam A. Manary, Nichole R. Orton, Kyle J. Boyle, Jingwen Hu

**Affiliations:** University of Michigan, Transportation Research Institute, Ann Arbor, MI 481095, USA; mmanary@umich.edu (M.A.M.); nritchie@umich.edu (N.R.O.); kjboyle@umich.edu (K.J.B.); jwhu@umich.edu (J.H.)

**Keywords:** wheelchair, automated vehicles, standards, occupant restraint, wheelchair tiedowns

## Abstract

This literature review summarizes wheelchair transportation safety, focusing on areas pertinent to designing automated vehicles (AVs) so they can accommodate people who remain seated in their wheelchairs for travel. In these situations, it is necessary to secure the wheelchair to the vehicle and provide occupant protection with a Wheelchair Tiedown and Occupant Restraint System (WTORS). For this population to use AVs, a WTORS must be crashworthy for use in smaller vehicles, able to be used independently, and adaptable for a wide range of wheelchair types. Currently available WTORS do not have these characteristics, but a universal docking interface geometry and prototype automatic seatbelt donning systems have been developed. In the absence of government regulations that address this situation, RESNA and ISO have developed voluntary industry standards to define design and performance criteria to achieve occupant protection levels for wheelchair-seated passengers that are similar to those provided by conventional vehicle seats.

## 1. Overview

For people with disabilities who do not drive, automated vehicles (AVs) would provide a welcome opportunity for independent travel. According to the American Community Survey (ACS), the overall percentage of people with a disability in the United States of America (USA) in 2017 was 12.7% [1]. Among the six categories of disabilities identified by the ACS, the highest prevalence across all ages was the 6.9% reported as having an ambulatory disability, which increases rapidly with age. In the 2017 National Household Travel Survey, 25.5 million people over age five report disabilities that limit their ability to travel [2]. Of these, 11.6% use a manual wheelchair, 3.9% use power wheelchairs, and 4.4% use scooters, indicating that about 5 million people use wheeled mobility devices in the USA.

The Americans with Disabilities Act [3] and its interpretation as the ADA Accessibility Guidelines [4] through the US Access Board provides detailed transportation requirements that are translated into regulations by the US Department of Transportation. These establish necessary minimum levels of accessibility and accommodations that are required in compliant public transportation, including requirements for assistance by a driver or other operator. However, these requirements do not consider the scenario where an individual with a disability transported in a public vehicle without a driver or other operator.

This paper reviews the literature related to wheelchair transportation safety, with a focus on topics that are relevant for providing the opportunity for safe, independent use of automated vehicles to people who use wheelchairs.

## 2. Wheelchair Transportation Safety Basics 

A best-practice travel recommendation for individuals who use wheelchairs and travel in passenger vehicles is to transfer to the original production vehicle seats and make use of the vehicle’s occupant protection systems [5]. An AV intended to be used in this way would also need an automated method of stowing and securing a wheelchair and transfer aids.

People for whom transfer from their wheelchairs is infeasible or impractical can use adapted vehicles, which are configured to allow use of a wheelchair as vehicle seating. Vehicle modifications include increasing cabin height by 20–25 cm inches, ramps or lifts to facilitate ingress and egress, adaptive controls for those who can drive, specialized hardware to secure the wheelchair to the vehicle, and a method of protecting the occupant in a crash that is compatible with wheelchair use. The last two elements are commonly referred to as the Wheelchair Tiedown and Occupant Restraint System (WTORS). In addition, people using wheelchairs as vehicle seating should choose wheelchair, WTORS, and accessories that have been crash tested according to voluntary guidelines prescribed in ANSI/RESNA Volume 4 WC19 (see Section 5).

## 3. Wheelchair Securement Systems

**Figure 1:**
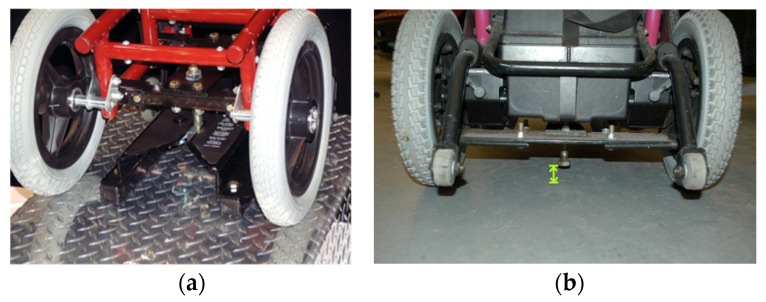
Examples of wheelchair engaged with docking system mounted to vehicle floor (**a**,**b**) and an example of mating hardware with low ground clearance (**b**).

To travel solo in a private vehicle while using wheelchairs as vehicle seating, individuals must firmly secure their wheelchair to the vehicle. The most common strategy for people who drive while seated in a wheelchair involves customized hardware added to the bottom of wheelchair that docks into a securement system mounted on the vehicle floor. An example is shown in Figure 1. Good function of these systems depends on maintaining close alignment of the mating hardware to allow effective docking to occur between the wheelchair and vehicle. However, day-to-day differences in wheelchair tire pressure or added wheelchair cargo can be enough to obstruct the process. A recent study showed that half of users needed multiple attempts to dock their wheelchairs in these securement systems [6]. In addition, this design of wheelchair docking system often reduces the ground clearance of the wheelchair and increases the difficulty of traversing over door thresholds and uneven surfaces. Such systems are also customized for a particular pairing of a single user and a single private vehicle and are not adaptable to a shared AV paradigm where one wheelchair space needs to accommodate many different people using wheelchairs.

**Figure 2:**
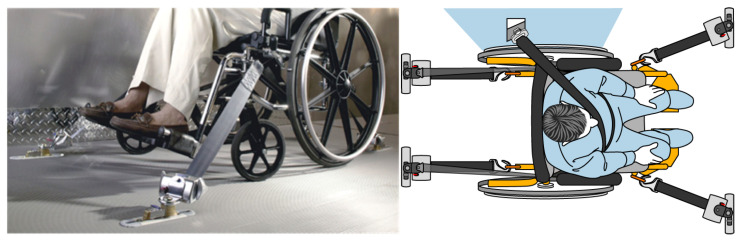
Example of a wheelchair secured by 4-pt strap tiedown system.

For people traveling as passengers in modified vehicles or via public transportation, the most common method of securing the wheelchair to the vehicle is a 4-point strap tiedown system. An example is shown in Figure 2. With this tiedown method, four straps are anchored to reinforced points on the vehicle floor and hooked onto the wheelchair. This system allows a single WTORS to secure a wide range of wheelchair types and has been shown to be very effective in the field. Although this system is the most common travel scenario for people who remain in their wheelchairs, few wheelchair users can independently secure their own wheelchairs with this technique. This system also usually requires a third party to assist with the application of the seatbelt and its routing around wheelchair features. Often this means that a person the wheelchair user is unfamiliar with must enter personal space and physically contact them to apply the seatbelt. This can be uncomfortable for wheelchair users. On a public transportation system, the person providing assistance is often the driver of the vehicle, which necessitates a longer dwell time at the stops where the wheelchair user boards and alights from the vehicle, impacting the timeliness of the transit schedule. Because a caregiver or driver may not be present to help, this approach is not a viable solution for AVs.

**Figure 3:**
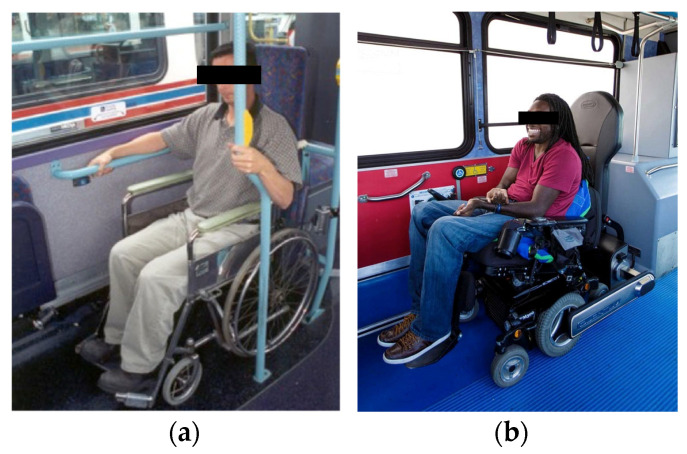
Rear-facing wheelchair passenger station (**a**) and Q’Straint Quantum automated docking system (**b**).

ADA guidelines allow the use of rear-facing wheelchair passenger stations on large, heavy buses. Examples are shown in Figure 3. These offer a high level of independence on large accessible transit vehicles (LATVs), and provide wheelchair users with a similar level of protection as the other LATV passengers who are not restrained in the bus seats and who are allowed to stand during vehicle travel. However, rear-facing stations are not robust enough to pass the crash severity requirements for lighter, minivan-sized vehicles. Rear-facing wheelchair passenger stations have been deployed since 2005 in some major metropolitan bus systems, while the new Q’Straint Quantum autonomous docking stations (an enhanced version of rear-facing station) have entered the market more recently.

LATVs will always have a lower range of crash severities compared to passenger vehicles primarily because of their higher mass, but for lighter-weight AVs, both protection in high severity crashes as well as independent use are needed. The operating speed of an AV will influence the overall distribution of crash severities, but even an AV that is unlikely to cause a crash can experience a high severity crash because other vehicles could strike it at high speed. In addition, initial deployments of AVs allow emergency braking levels of up to 1 g, whereas emergency braking levels of human drivers rarely exceed 0.4 g. Higher levels of braking could lead to injury for a passenger in an unsecured wheelchair.

**Figure 4:**
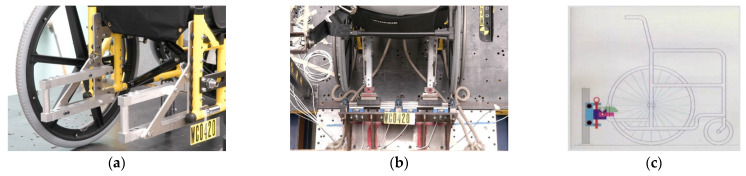
UDIG attachment hardware on wheelchair (**a**), a wheelchair engaged with UDIG mounting hardware (**b**), and a diagram of UDIG geometry (**c**).

**Figure 5:**
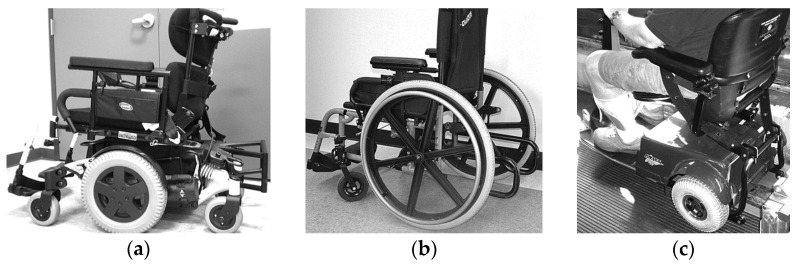
A power wheelchair (**a**), manual wheelchair (**b**), and scooter (**c**) equipped with UDIG-compatible hardware.

The Universal Docking Interface Geometry (UDIG), shown in Figure 4 [7], is one proposed solution for making docking stations that can work in a public transportation setting where one wheelchair station must secure many types of wheelchair users. UDIG defines an interface geometry and interface location that can be the basis for design of docking stations and dockable wheelchairs. For the wheelchair, as shown in Figure 5, the required UDIG elements are two 22-mm diameter, 75-mm long, vertical tube-shaped features located on the lower rear of the wheelchair that are spaced 222 to 333 mm apart. These are located on the wheelchair so that the bottom of the tubes is 203 mm above the floor surface. If needed to control rotation performance during impact, the UDIG can also include a horizontal bar that connects the tops of the two vertical tubes and is 319 mm above the floor. This concept is akin to the standardization of trailer hitches that allow any semi-tractor driver to attach and tow any trailer. The UDIG design has the advantage of not decreasing ground clearance on equipped wheelchairs. Although this geometry has been defined, prototyped, crash tested, and field tested [7,8,9,10], it has not been incorporated into any commercial products to date. However, specifications for UDIG geometry are included in informative and normative annexes of current wheelchair transportation safety standards in the US and internationally. The key barrier to implementation of the UDIG system is the voluntary nature of wheelchair safety standards; vehicles must be equipped with UDIG docking hardware and wheelchairs must be equipped with UDIG securement hardware before the system is feasible.

Hobson and van Roosmalen [7] describe the development and testing of an automatic docking device meeting UDIG specifications for public transit vehicles. The system was crash tested using WC-18 specifications. An energy-absorbing component was evaluated in one test but did not provide additional benefit. The researchers received input on the system from a focus group of wheelchair users, which led to refinements of the maneuvering area, user controls, driver controls, and emergency release mechanisms for installation on a large transit bus for usability testing. Initial evaluation was performed on a test track with one user evaluating the ride performance of a manual and power wheelchair secured with the docking station. Displacement was monitored during braking and turning maneuvers. The manual wheelchair had more than 50 mm (2 in) of displacement allowed by ADA requirements during turning, but other conditions were acceptable. Because the system was evaluated on a large transit bus, an occupant restraint was not included in the evaluation.

**Figure 6:**
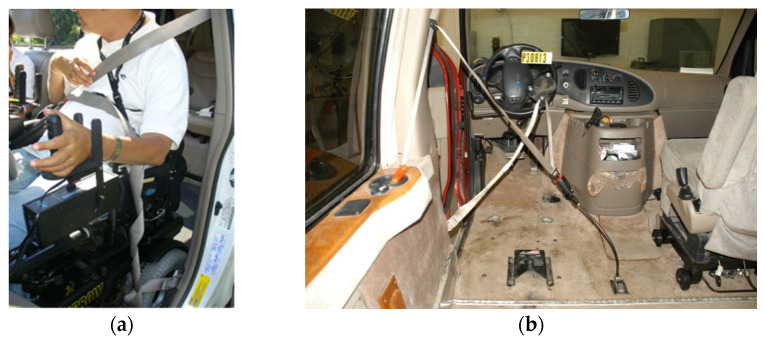
Driver using vehicle belt with poor fit (**a**) and a “drive-in” belt arrangement (**b**).

A subsequent study compared usability, comfort, and independent use of 4-point strap tiedown, a rear-facing station, and a UDIG compatible auto-docking system on a large transit vehicle [9,10]. Twenty subjects who could transfer to the modified wheelchairs took a 15-min bus ride using each device and then completed a survey. Figure 6 shows the three types of wheeled mobility devices used in the study, each equipped with hardware that meets the UDIG specifications. Participants rated the autodocking and rear-facing stations as being faster and easier to use than the 4-point strap tiedown system. Discomfort from riding rear-facing, as well as the inability to see stops, was commonly reported for the rear-facing station. Fourteen occupants preferred the autodocking station for travel, because it allowed secure and independent use. However, they noted that requiring specialized hardware on the wheelchair was a barrier to use. All securement systems met the less than 50 mm displacement requirement established by the ADA. Maximum occupant acceleration during maneuvers was 0.76 g.

The effect of UDIG hardware placement on a wheelchair was explored using DYNAMAN models [11]. They used a model of a manual wheelchair equipped with a wheelchair-mounted lap belt and a vehicle-mounted shoulder belt. They varied the fore-aft location, lateral spacing, and vertical spacing of the wheelchair-mounted UDIG hardware by 100 mm to evaluate effect on wheelchair and occupant (midsized male ATD) kinematics. They simulated front impact with a 30 mph/20 g pulse, and side impact with a 15 mph/12 g pulse. The center of gravity (CG) of the wheelchair was varied from 30 cm to 45 cm above the floor. Reported outcomes were frontal or lateral excursions. The lowest excursions in frontal impact occurred when the UDIG was mounted high, wide, and forward, in wheelchairs with a low CG. The lowest excursions in side impact occurred with the widest UDIG mounting configuration.

Table 1 summarizes the different types of existing wheelchair tiedown systems according to their independent use, crashworthiness level, and compatibility between different wheelchairs and vehicles. Currently, only a system meeting UDIG requirements allows independent use in large and small vehicles using any combination of wheelchair and vehicle equipped with appropriate hardware that would be needed in an AV.

## 4. Belt Restraint Systems Used with Wheelchairs

People who drive while seated in their wheelchairs in private vehicles often use the lap and shoulder belt restraint system provided with the vehicle. However, in these modified vehicles, the inboard buckle is often mounted to a stalk attached to the floor, as the original vehicle inboard buckle has been removed with the vehicle seat to create the wheelchair station. In addition, active features such as seatbelt pre-tensioner and occupant classification system may be disconnected as part of the vehicle modification. If the driver’s dexterity will not allow them to buckle a seatbelt, an alternative approach is to drape the pre-buckled lap and shoulder belt onto the steering wheel so that the driver can maneuver into the restraint while seated in the wheelchair. This option often results in a loose belt restraint or poorly placed belts due to interference with the wheelchair armrests and controls. Examples are shown in Figure 6.

**Figure 7:**
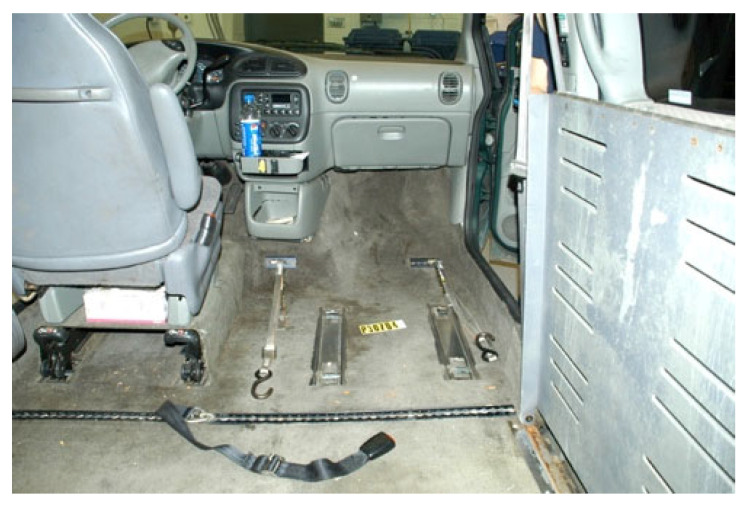
Passenger belt system.

An example of a restraint systems used for passengers traveling in wheelchairs is shown in Figure 7. Because a wheelchair user who depends on a 4-point strap tiedown system to secure the wheelchair will likely need assistance, the belt restraint systems are also designed to be donned with assistance. They may or may not include a retractor.

A past study evaluated the wheelchair securement and occupant restraint systems of 29 individuals who drove (*n* = 21) adaptive vehicles or traveled in private vehicles while seated in wheelchairs (*n* = 8) [6,12,13]. This study evaluated the ease of ingress and egress, wheelchair securement, and seatbelt use as well as the locations of seatbelt anchor points and other vehicle interior features through observation of volunteers and subject surveys. The posture and position of the wheelchair user in their preferred travel position was also quantified. Although recommendations for placing seatbelt anchors to provide optimal protection had been available for some time, and the process of modifying a vehicle even allows customization for a particular size of person using a wheelchair, almost none of the participants had good seatbelt fit. Many individuals also had trouble using the seatbelt systems. Several subjects needed torso support to maintain a driving position, which led to modifications of the belt restraint system that compromised belt fit. Despite the poor belt fit documented in this study, most of the subjects indicated that they felt safe using their wheelchair tiedowns and occupant restraint systems. Seven of the twenty-one drivers had deactivated steering-wheel airbags.

Wheelchair features, such as closed-front arm supports and lateral thigh support features, often prevent good fit of the seatbelt system to the rider. The ANSI/RESNA voluntary wheelchair standard for wheelchairs used as seats in motor vehicles, ANSI/RESNA Volume 4: Section 19 (commonly called WC19), includes wheelchair performance requirements to eliminate these conflicts. People with dexterity, range of motion, and vision deficits often have difficulty buckling, applying and releasing conventional seatbelt systems.

Poor fit of safety systems for people seated in wheelchairs, along with higher levels of non-use and misuse of seatbelts, have also been documented in analysis of field injury events [14,15]. In this study, in-depth investigations of 69 incidents involving 74 occupants seated in wheelchairs were reviewed. Most of the incidents were frontal crashes, although three non-crash events were included. Eighty-one percent of occupants were appropriately using tiedown systems, and only one case had a failure. However, only 29% of the occupants were appropriately restrained by a lap-shoulder belt; lack of use and misuse that resulted in poor belt fit were frequent. Sixty-two percent of occupants in these cases experienced serious injury, with 10 cases resulting in death.

**Figure 8:**
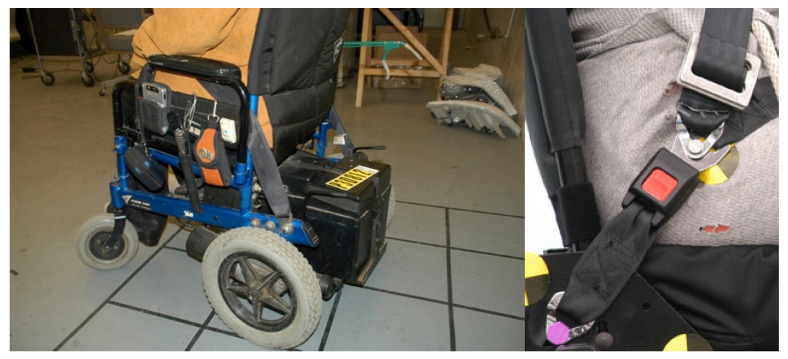
Crash-tested wheelchair restraints.

Because of challenges in donning belt systems, as well as issues with fit, an alternate solution is for the wheelchair to be equipped with a crash-tested belt restraint system. Examples are shown in Figure 8. These belt systems are currently offered on a limited number of wheelchairs and the option must be offered on a WC19 compliant wheelchair. While requirements to include crash tested belt restraint systems on all wheelchairs would simplify use of AVs and likely improve belt fit (thus increasing crash protection) for all occupants, the voluntary nature of wheelchair testing standards coupled with the increased expense of equipping wheelchairs with crash-tested belt restraints has limited their widespread deployment.

**Figure 9:**
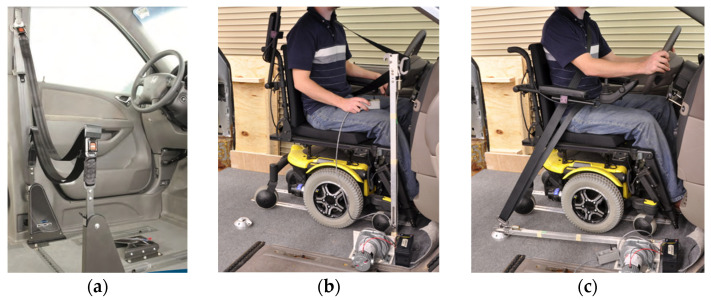
Q’Straint DIOR self-donning belt system (**a**) and UMTRI prototype belt donning system (**b**,**c**).

Some previous work explored the concept of a self-donning seatbelt. Q’Straint developed the DIOR system shown on the left in Figure 9, but has discontinued its sale. A prototype donning system, shown on the right two pictures of Figure 9, has been developed in a past research project [16]. The seatbelt deployment system (SBDS) uses the vehicle equipped seatbelt, but with the buckle mounted to a rigid rotating stalk. The length of the stalk can be adjusted for the size of the occupant and wheelchair so the side-view lap-belt angle falls within the recommended 45 to 75 degree range relative to horizontal. The wheelchair user moves into the seating position, then uses a hand control to rotate the buckle down to the floor, where it is secured in a floor-mounted anchorage pocket. The SBDS works best with wheelchairs that have an open-front arm support design. The system has successfully been crash tested using WC19 procedures.

## 5. Relevant Standards

The situation where a person uses a wheelchair as a seat in a motor vehicle is not completely addressed in US federal motor vehicle safety standards (FMVSS), but groups of stakeholders have used the precedents and crash protection principles of the FMVSS to establish voluntary industry standards for this circumstance. The Rehabilitation Engineering Association of North America (RESNA) has a suite of standards contained in four volumes that establish ways to measure, define, and test wheelchairs and wheelchair components, including Volume 4 that currently is comprised of four sections: Section 10 Wheelchair Containment and Occupant Retention Systems for use in LATV, Systems for Rear-Facing Passengers (WC10), Section 18 Wheelchair Tiedowns and Occupant Restraint Systems (WC18), Section 19 Wheelchair used as Seats in Motor Vehicles (WC19), and Section 20 Wheelchair Seating (WC20). A set of similarly intentioned standards exist for global use within the International Organization for Standardization (ISO) that are developed and maintained by international experts in a working group under Technical Committee 173, Subcommittee 1, Working Group 6. These ISO standards overlap significantly with the North American standards, with standards 10865-1, 10542-1, 7176-19, 16840-4, being international versions of WC10, WC18, WC19, and WC20, respectively. The set of ISO standards also includes 10865-2 that specifically addresses wheelchair spaces in LATVs for forward-facing passengers and places a high emphasis on independent use. These standards currently include test protocols for frontal and rear impacts only that were developed and modified using results of multiple test programs [5,17,18,19,20,21,22,23,24].

### 5.1. WC10

WC10 [25] provides specifications and test procedures for rear-facing wheelchair passenger stations (RF-WPS) that are intended for use only in large accessible transit vehicles (LATV). For LATVs that have lower crash rates per mile, as well as lower severity crashes because of their larger mass, providing a passive containment system for wheelchair users is sufficient to provide a reasonable level of transportation safety with a higher degree of personal independence. The level of safety is comparable to unrestrained seated passengers or standing passengers who hold onto stanchions or straps to resist movement during travel.

Part 1 of the standard describes the scope, relating to RF-WPS, while part 2 references other RESNA and federal standards and part 3 provides relevant definitions. Part 4 defines design requirements in terms of the needed geometry and features required for a RF-WPS, which are described in more detail in Annex A. Performance requirements found in part 5 include testing the static strength of excursion barriers with methods found in Annex C, the allowable amount of wheelchair excursion for the wheelchair when tested using procedures found in Annex B, and a required coefficient of friction for the flooring material. Part 6 contains information, labeling, and instruction requirements, while Part 7 contains reporting requirements. Annex D defines specifications for a manual surrogate wheelchair (MSWC) and a scooter surrogate wheelchair (SSWC) that can be used to evaluate the RF-WPS. Annex E contains design guidelines for RF-WPS.

### 5.2. WC18

WC18 [26] applies to wheelchair tiedown and occupant restraint systems (WTORS), consisting of a system or device for securing wheelchairs, and a system of belts for restraining occupants seated in wheelchairs. This includes both strap-type and docking-type securement systems. The standard is focused on the application of WTORS to passenger vehicles so assumes a more severe crash environment. Part 2 references other RESNA standards and federal motor vehicle safety standards; definitions are included in Part 3.

Part 4 of WC18 defines design requirements. They define what elements comprise a complete WTORS system, requirements for wheelchair tiedowns and securement devices, specifications for wheelchair tiedowns/securement adaptors, and features and relevant federal compliance requirements for occupant restraint components.

**Figure 10:**
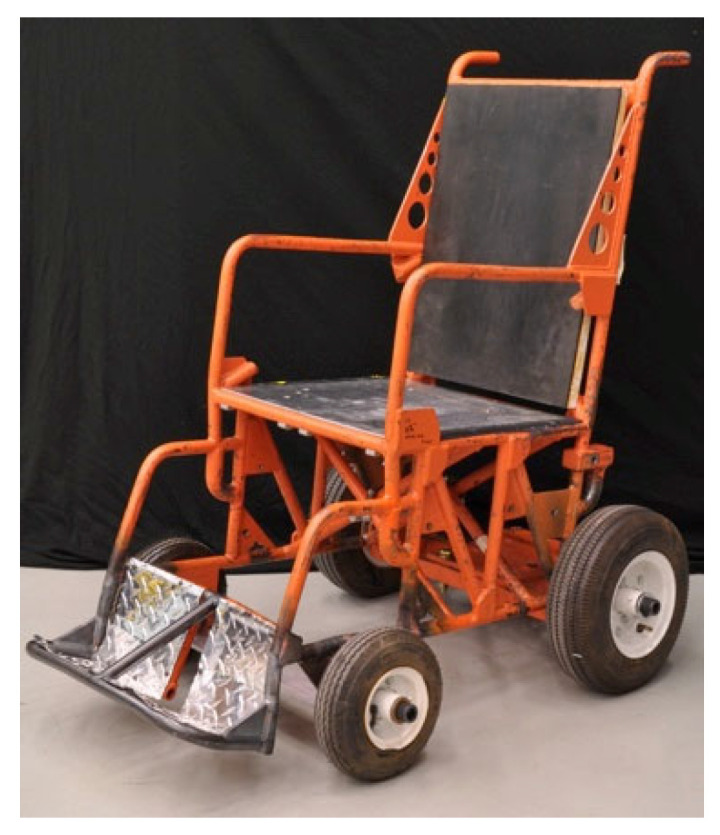
Surrogate wheelchair fixture.

Part 5 lists performance requirements. WTORS must meet flammability requirements of FMVSS 302, as well as most requirements of FMVSS 209. If the WTORS has a lap-shoulder belt component, crashworthiness in frontal impact is assessed in two tests, one with the lap-shoulder belt anchored to the vehicle, and one that uses the surrogate lap-shoulder belt with a wheelchair-anchored lap belt defined in Annex D of WC19. Options for testing with different combinations of belt restraints are also included. Tests are conducted either with a specific wheelchair model (SWM) or with a surrogate wheelchair pictured in Figure 10 and defined in Annex E. Sled test procedures use a 48 km/h (30 mi/h), 20 g acceleration pulse, similar to that used in FMVSS 213 for frontal impact testing, although the allowable corridor is wider than the FMVSS 213 pulse. Details regarding the test buck, instrumentation, ATD positioning, wheelchair preparation, pre and post-test measurements, and reporting requirements are also included in Annex A. To pass the test, the system must meet wheelchair, head, and knee excursion limits specified for the ATD used in the test. Values are provided for 3YO, 6YO, 10YO, 5th female, 50th male, and 95th male ATDs. In addition, the ATD must be seated in an upright position after the test, the WTORS components should not completely fail, and the wheelchair should remain undamaged if a SWM is used in the test.

WTORS performance requirements also include geometric and adjustability specifications that are evaluated using procedures in Annex B. Annex C includes procedures for assessing the performance of WTORS under partially engaged conditions. WTORS must have less than 25 mm of slip when tested under conditions described in Annex D. WC18 has requirements regarding written materials, including product identification and labeling, instructions for installers, advice and warnings for installers, user and maintenance instructions and warnings, in-vehicle placards, and instructions for WTORS components and subassemblies sold separately. The last part provides direction on how to document compliance with the standard. In addition to the annexes that describe test procedures, Annex F provides design and performance recommendations.

### 5.3. WC19

The scope of WC19 [27] “is to establish design and performance requirements, and associated test methods, for wheelchairs related to their use as seats in vehicles”. Part 2 of the standard references multiple federal motor vehicle safety standards, as well as related RESNA voluntary standards. Part 3 provides definitions of terms used in the standard. Part 4 specifies design requirements related to seated posture, mass, size, turning radius, and head/back support, reduction of sharp edges, securement points for four-point strap tiedowns, and wheelchair-anchored belt restraints. The design requirements for the securement points specify the geometry and locations of the four securement points and how they should be attached to the wheelchair; Annex G provides recommendations on securement point design. Wheelchair anchored belt restraints should provide a side-view lap belt angle of 30 to 75 degrees (45 to 75 is preferred) relative to horizontal, and Annex H provides belt restraint design recommendations. The belt restraint specifications also define a level of adjustability and attachment hardware for connecting to a vehicle-mounted shoulder belt.

**Figure 11:**
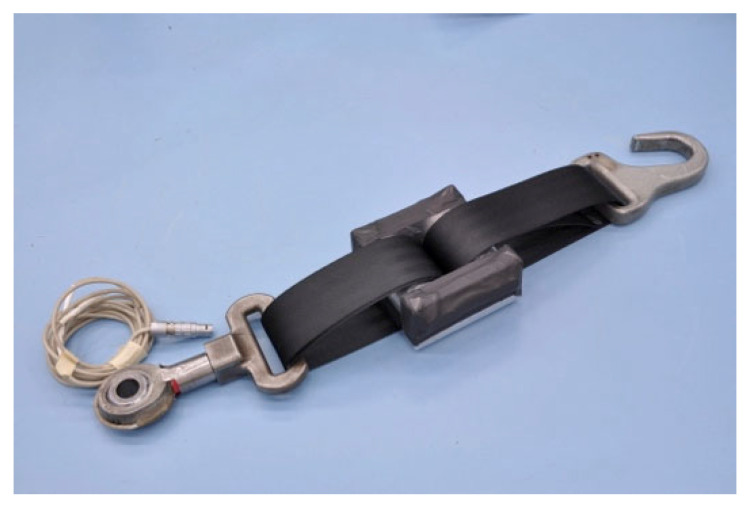
Surrogate WTORS.

Part 5 of WC19 describes performance requirements. Tiedown hooks must be able to be engaged to wheelchair securement points with one hand. Seatbelt components must comply with requirements of FMVSS 209 and/or FMVSS 213. Frontal-impact crashworthiness is assessed with the wheelchair secured by a surrogate four-point strap tiedown system (defined in Annex D and shown in Figure 11), using an adult or pediatric anthropomorphic test device (ATD). To pass the crashworthiness test, wheelchair components must not fail, and the securement points cannot deform to the point where the tiedowns cannot be removed. The wheelchair must be upright and the ATD must be in a seated posture post-test. Maximum wheelchair, knee, and head excursion limits are specified for ATDs ranging from the 3YO to 95th percentile male. Annex A describes the frontal-testing impact procedures, which are essentially the same as the procedures defined in WC18.

Additional performance requirements specify that there must be clear paths that are free of sharp edges for the four-point strap tiedowns to reach the securement points. Test procedures for assessing access are provided in Annex B. Lateral stability is assessed with a tilt test described in Annex C. Turning radius must be measured using procedures from RESNA WC:1, Part 5, and included in product literature. Wheelchairs must receive ratings of acceptable or higher regarding accommodation of vehicle-mounted lap-shoulder belt systems, evaluated using procedures in Annex E. The procedures assess ease of achieving proper belt placement on the ATD, lap belt contact and location, shoulder belt contact and location, lap belt angle, lap belt path clear path to anchor points and proximity to sharp edges. 

Part 6 of WC19 specifies requirements for product labeling and wheelchair manufacturer literature. Requirements are included for identification and labeling, presale literature, user instructions, and user warnings. Part 7 specifies how to document compliance with the standard.

Annex F of RESNA WC19 provides specifications for the universal docking interface geometry (UDIG), while Annex I provides information about obtaining other standards referenced in Part 2.

### 5.4. WC20

While many wheelchairs are produced as a single piece of equipment made by one manufacturer, for others, a wheelchair base from one manufacturer can be paired with different styles of seating systems made by another company to better accommodate the specific needs of the person using the wheelchair. WC20 [28] was developed to allow evaluation of the crash performance of different seating systems independent of the wheelchair frame. Seating systems consist of a seat, back support, and attachment hardware. Part 1 of the standard defines the scope, Part 2 incorporates other references, and Part 3 defines terminology.

**Figure 12:**
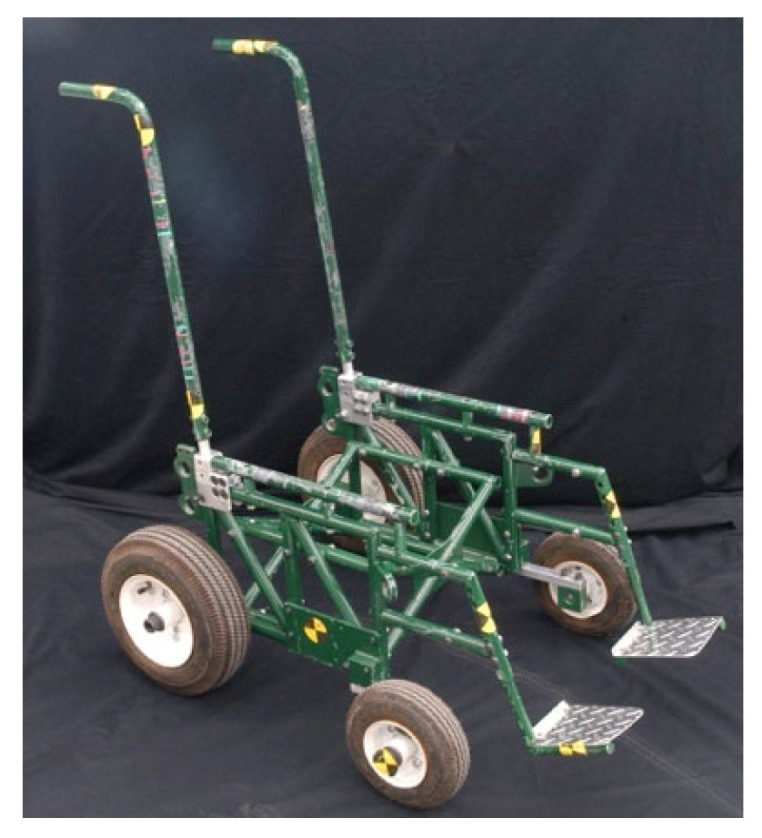
Surrogate wheelchair frame.

Part 4 describes design requirements related to sharp edges and accommodating vehicle-anchored belt restraints. Part 5 describes performance requirements, which are essentially the same as those required in WC19 for frontal crashworthiness and accommodation of vehicle-mounted belt restraints. However, testing of wheelchair seating systems is performed using a Surrogate Wheelchair Base (SWCB) defined in Annex B and shown in Figure 12. The SWCB allows evaluation of different styles of seating systems independently, and allows lateral adjustability to accommodate smaller and larger wheelchair seating systems.

Parts 6 and 7 describe requirements for written materials and documentation similar to those found in WC19. In WC20, Annex A describes frontal-impact test procedures, Annex B specifies the SWCB fixture, Annex C contains method for evaluating accommodation of belt restraints, and Annex D contains methods for performing quasi-static tests of wheelchair seating systems (which are recommended before performing dynamic testing but are not required). Annex E provides sources for relevant information.

## 6. Wheelchairs and Side Impact

Since many wheelchairs are designed to fold along the centerline to facilitate storage, some wheelchairs that pass frontal impact testing standards may not demonstrate the same integrity during side impact crashes.

As part of the research funded by a NIDILRR Rehabilitation Engineering Research Center, side impact performance of occupied wheelchairs was explored. The work evaluated the current level of side impact crash protection afforded to wheelchair users seated in wheelchairs that comply with WC19 when secured with WTORS that comply with WC18. Since injury protection in nearside crashes is primarily addressed with vehicle features (padding, airbags, sidewall features), the work focused on farside crash protection, where features of the wheelchair and occupant restraint can improve occupant protection. The work considered three crash severities that were precedents in side impact protection at that time: an FMVSS 214 pulse for a van (14 mph/16 g), The EuroNCAP small vehicle pulse that is also used as a side impact pulse for CRS testing (15.5 mph/13 g) and the early US ANPRM for CRS side impact testing (14.5 mph/20.6 g and 21 mph/26 g). Manary [29] reported on the first phase of testing three side impact tests performed with the midsized male Hybrid III ATD in commercial wheelchairs secured with a 4-point strap tiedown systems. Test severities ranged from 23 km/h, 16.4 g to 30 km/h, 15.8 g. Two tests were performed with the shoulder belt in a far-side configuration, while the third test evaluated a near-side configuration without intrusion. The tiedown system was effective at limiting wheelchair movement to no more than 254 mm of excursion, and there was minimal deformation of the three wheelchairs that met WC19 requirements for frontal testing. The ATD moved out of the belt in the two far-side conditions, with excursions approximately double the excursions measured in the nearside condition, where the shoulder belt prevented the ATD from moving excessively laterally. The work continued with 6 more tests of manual, power, and stroller type wheelchairs, including one secured using UDIG. The wheelchair frames were well-secured by the WC18-compliant WTORS, including UDIG. However, the ATD was not well restrained from excursion when the upper shoulder anchor point was opposite the impact directions. In these cases, the lateral features of the wheelchair were heavily loaded by the ATD, and the ATD was not contained in a seated position. Wheelchairs equipped with fabric seating that hammocks the occupant did a better job of limited lateral motion than those with planar seating.

## 7. Vehicle Modifications

Information about modifying personal vehicles (typically vans) can be found on the National Mobility Equipment Dealers Association (NMEDA) national website as well as those from individual NMEDA dealers. 

Under the topic of safety, the NMEDA website states “Having the right type of equipment installed in a wheelchair accessible vehicle can not only transform your life with added mobility and independence, it can also prevent serious injuries caused by standard highway equipment.” This statement reflects the typical practice to disable airbags when modifying a vehicle for use by a driver seated in a wheelchair; current guidelines allow but do not require this practice [30]. While this recommendation was reasonable when airbags were first introduced and had a higher potential for inducing injury to occupants sitting too close to the steering wheel, this practice may no longer be warranted. Vehicle safety system engineers now design less aggressive airbags to work in an integrated manner with seatbelts that can include advanced features such as load limiters and pre-tensioners. Disabling the airbag in an adaptive vehicle may also disable the sensing systems used to activate the seatbelt features, reducing protection even further for these drivers. Sensors needed to control safety features may also be removed when vehicle seats are replaced with wheelchair docking stations.

The first section of NMEDA guidelines provides instruction to modifiers on how to document compliance with the Exemption to the Make Inoperative Prohibition (49 CFR 595.7). Specific modifications relative to occupant protection that are allowed include:

- 201u: Exemption if the roof is raised or the floor is lowered; pillars and roof rails around a ramp/lift are exempt if the floor and roof are not modified.
- 202: Person in a wheelchair is allowed to travel without rear head restraints
- 203: exempt because control devices often attached to steering wheel
- 204: exempt from displacement requirements in case modifications to the column are needed to install alternate controls
- 208: can remove/deactivate all airbags for front seating positions if a Type 2 or 2A seatbelt is installed in that position.
- 207, 214: can remove vehicle seat and exempt from side impact protection.

The remaining topics covered by NMEDA guidelines are summarized below, listing titles for each section and selected excerpts related to occupant protection. Of the forty different sections in the guidelines, only Section 26 addresses wheelchair and scooter securement and occupant restraint.

1. Consumer Documentation
2. General Best Practice. 3.18 specifies that “All mobility dealer installed lap belts will cross the occupant at the H-point.”
3. Service Practice (related to training and customer service). This section refers to fitting of seat belts and tiedowns as follows: “Of special note for drivers using adaptive equipment, a mid-conversion and final fitting with the end user or client present is expected to occur at the dealer location to fine tune equipment adjustments, determine tie-down locations, torso belt dimensions, etc”.
4. Vehicle Weight Ratings (how to calculate after the modifications are made)
5. General Electrical Specifications
6. High Tech and Low Tech Adaptive Equipment Definitions
7. Accelerator, Brake, and Clutch Pedal Modifications
8. Automotive Wheelchair Roof Carriers/Loaders
9. Driver Training Brake (installed for use when a driver seated in a wheelchair is first learning how to operate the vehicle).
10. Electrically Powered Seat Bases, where a vehicle seat is replaced by another seat with greater maneuverability that would allow a person to transfer from a wheelchair docked in an adjacent seating position.
11. Extended doors
12. Exterior Door and Lift Controls
13. Floor lowering. This section states “When installed in the driving position, the seat shall be located so as to allow the driver to use the OEM seat and shoulder safety belt system.”
14. Left foot accelerator control
15. Mechanical Hand Controls
16. Parking Brake
17. Power Door Openers
18. Raised Roof. This section includes a statement regarding strength of upper belt anchorages. “If a NMEDA raised roof F/CMVSS 210 manual exists for the vehicle make and model year to be modified, the manufacturing instructions must be followed or the modifier must document their pathway to F/CMVSS compliance with a prototype vehicle test report for the upper seat belt anchorages under F/CMVSS 210.”
19. Seats. This section states: “Seat belt geometry must be maintained within OEM specifications.”
20. Steering Column Extension
21. Steering Wheel Devices. This section states: “If interference with operation of the airbag cannot be avoided the airbag should be deactivated while the steering device is in use.”
22. Transfer Aids. This section states: “Transfer aids shall not be installed to interfere with the function of the vehicle’s airbag systems.”
23. Vehicle Steering Column Mounted Accessory Controls
24. Unoccupied Lifts
25. Wheelchair and Scooter Securement
26. Wheelchair Flooring
27. Power Elevating Platform for Wheelchair Driver. This section includes specifications for seat belt installation as follows: “There shall be a three point seat belt provided for the wheelchair occupant. If the seat belt is anchored to the elevating platform, it shall be tested as per F/CMVSS 210 in conjunction with the requirements of Section 28.7. If the seat belt is anchored to the vehicle floor, it shall be tested as per F/CMVSS 210 independently of the load requirements of Section 28.7.”
28. Backup Braking System
29. Reduced Effort Hydraulic Steering System and Backup Hydraulic Steering System
30. Electronic Vehicle Interface
31. Gear Shifter Operation
32. Horizontal Steering System
33. Power and Gas Brake System
34. Reduced Effort Braking System
35. Reduced Effort Electronic Power Steering System and Electronic Power Steering Backup System
36. Remote Steering Systems
37. Secondary Control/Systems. This section states: “Installation of the controls shall assure the greatest possible retention of OEM driver and occupant protection features including collapsible steering column, knee bolsters and airbags.”
38. Interlocks
39. Off-Site Installation and Service Policy
40. Hybrid/Electric Vehicles

Appendix A: Summary Descriptions of FMVSS/CMVSS

Appendix B: Out of Service Area Agreement for NMEDA Dealers

Appendix C: Adaptive Equipment Transportation Industry Terminology

Appendix D: Labels and Descriptions

Recent computational modeling studies performed demonstrate that modern airbags are more likely to improve protection than cause injury in frontal crashes (32). When vehicles are modified to accommodate drivers or passengers using a wheelchair, the entire floor is removed and replaced with a lower reinforced floor. This is necessary to allow sufficient clearance for the occupant to enter the vehicle while seated in a wheelchair, which typically has a higher seating height than vehicle seats. In addition, rear passenger locations are often placed in the center of the vehicle, to allow greater room for the occupants to enter and maneuver their wheelchairs into position relative to the tiedown locations, as well as to provide space for a driver or caregiver to attach the 4-point strap tiedowns. As a result, the rear occupants are not usually situated to benefit from deployment of side curtain airbags.

## 8. Computational Modeling

Few studies of computational modeling for WTORS, wheelchairs, and wheelchair users have been published. Table 2 summarizes details of previous computational models related to wheelchair occupant protection. One of the first computational models of wheelchairs under frontal impact loading used DYNAMAN to model the surrogate wheelchair with a Hybrid III midsized male ATD [31]. Parameter studies with this model investigated effects of tiedown stiffness, height of tiedown attachment point on the wheelchair, wheel stiffness, and crash pulse severity at the lower and higher ends of the ISO corridor. Other early studies used DYNAMAN to model a commercial power wheelchair, and then to estimate how variations in seat and seatback stiffness and angle affect kinematics during frontal impacts [32,33]. Subsequent studies developed a MADYMO (TASS International Software and Services, Helmond, Netherlands) model of a commercial manual wheelchair (Invacare Compass Allegro) validated against tests using the surrogate WTORS and a Hybrid III midsized male ATD [34]. Other researchers performed additional simulations using this wheelchair model with small female, midsized male, and large male occupants seated to evaluate different lap belt angles [35,36,37]. They recommend a range of 45 to 60 degrees as best for accommodating a range of occupant sizes using wheelchairs as seating. This model was also validated under rear impact conditions [38].

More recently, a full set of MADYMO models, including a surrogate wheelchair, docking or 4-point tie-down system, 3-point seatbelt, knee bolster, steering wheel, and driver airbag, was developed to investigate restraint system designs on protection for wheelchair users [15,39]. These models have been validated against multiple sled tests with varied ATD sizes, belt fit, and airbag conditions. The parametric simulation results clearly demonstrate that wheelchair-seated occupants without a seatbelt or a seatbelt with poor belt fit experience higher injury risks in frontal crashes. The simulation studies also demonstrated that a properly deployed driver airbag can provide important safety benefits for occupants with a wide range of sizes who are seated in wheelchairs in frontal crashes. Therefore, optimizing the seatbelt system for wheelchair users should consider a restraint system for frontal impact that includes airbags. No models of wheelchairs in side impact conditions have been developed to date.

NHTSA performed crash testing in two vehicles equipped with wheelchairs in the driving position [40] to validate modeling performed at UMTRI [39]. Tests were performed with modified 2015 Dodge Caravans, with Q’Straint QLK-150 docking stations securing Quantum Q6 2.0 wheelchairs. A mid-sized male ATD was positioned to represent the average posture documented in a study of wheelchair users [15]. One test was run with the driver airbag, and one without. Comparison of Injury Assessment Reference Values between the two tests showed that the airbag reduced injury risk to head and chest, while it remained the same for the neck, and showed an increase for femur loads (though they remained below critical levels). The back of the wheelchair failed from inertial loading.

## 9. Design Space

The Americans with Disabilities Act Accessibility Guidelines (ADAAG) for Transportation Vehicles specifies that a floor space measuring 760 mm (30 in) wide and 1220 mm (48 in) long is required to accommodate a wheelchair, based on common wheelchair dimensions found in the 1970s. The ADA requirements also specify that vehicle door heights should be at least 1422 mm (56 in). A detailed study of combined occupant and wheelchair dimensions was published in 2010 to quantify the space needed to accommodate a greater variety of wheelchair and occupant sizes [41,42]. In this study, they measured 276 people using manual wheelchairs, 189 using power wheelchairs, and 30 using scooters.

Statistical data on the range of occupied heights from this study are shown in Table 3. Occupied height is defined as the distance from the floor to the highest point on the person’s head. The mean value of power chair users is 25 mm higher than the mean value of manual chair users, while the mean value of scooter users is 47 higher than those of power users. The current ADA requirement for vehicle door height is 1422 mm (56 in), which would accommodate 95% of manual and power chair users, but not the upper range of scooter users.

The Steinfeld et al. study evaluated knee clearance of people using wheelchairs, with a suggested value of 700 mm (28 in) as suitable for accommodating 95% of occupants using wheelchairs. Their overall results regarding the percentile distribution of knee profiles across their range of subjects can provide guidance when designing knee bolsters or knee airbags. In addition, although variation in wheelchair seat heights is not explicitly reported, the range of locations where the lap belts should be placed across the range of occupants can be inferred from these data.

## 10. Accessibility and Automated Vehicles

The previous sections of the report focus on literature related to providing wheelchair users the opportunity to use automated vehicles safely, easily, and independently by developing an automated wheelchair tiedown and occupant restraint system. Several organizations have recently addressed broader needs of people with disabilities related to using automated vehicles, and a short summary of their efforts is reported in this section.

The Auto Alliance organized a 3-workshop series to address automated vehicles and accessibility, including legal and policy issues [43]. To ensure that AVs are accessible for people with disabilities, collaboration will be needed among users, vehicle manufacturers, AV designers, wheelchair manufacturers, assistive device manufacturers, and government agencies. They provide a summary of inclusive design considerations that could be a starting point for best practice guidelines for AV design, and recommend that people with disabilities be consulted throughout the design process. Additional regulatory guidance beyond that provided by the ADA and Access board would be useful. As seen earlier in Table 1, no current production wheelchair tiedown systems are suitable for use in AVs, and additional research is needed to develop a feasible automated WTORS. Wider use of crashworthy wheelchairs is limited by current policies regarding insurance reimbursement of transit features on wheelchairs.

Part of the Public Listening Summit on Automated Vehicles hosted by the US Department of Transportation [44] addressed disability and accessibility concerns. Clearer guidance on accessibility requirements for AVs are needed. They pointed out that different types of disabilities (vision, hearing, cognitive, or mobility) may require different accommodations. They noted that standardization of auxiliary mobility aides, such as wheelchair lifts or accessible displays, would facilitate use of vehicles where a human driver is not present.

The Intelligent Transportation Society of America published a report on *Driverless Cars and Accessibility: Designing the Future of Transportation for People with Disabilities* [45]. Fully automated vehicles offer people with disabilities new opportunities for independent access to employment, health care, and education. Deployment of AVs could potentially increase annual vehicle miles traveled substantially, as the American Association of Retired Persons (AARP) estimates that up to one-third of people in the US do not currently drive. AVs would be beneficial to people with temporary disabilities, and may allow older people to remain in their homes longer. While technologies are available that would allow people with different types of disabilities use an AV, standards and best practice recommendations would be welcome. Strategies for dealing with emergency situations is a key issue, as well as other non-driving tasks typically handled by a driver (ingress/egress, passenger monitoring.) Additional infrastructure is needed to accommodate people before and after they travel in an AV. Deployment of AVs may change the transportation system, reducing private vehicle ownership and increasing ride-sharing opportunities. Collaboration among a wide range of stakeholders will be needed to ensure that future transportation options are available to everyone.

A white paper discussing the impact that self-driving cars could have on the lives of people with disabilities [46] indicates that approximately 6 million Americans with disabilities have trouble accessing the transportation they need. Limited transportation options can result in reduced economic opportunities, isolation, and diminished quality of life. Improving transportation options for people with disabilities could lead to greater employment and substantial savings from fewer missed medical appointments. As automated vehicles are introduced to the fleet, service providers and manufacturers need to ensure that the needs of people with disabilities are considered in their design. This section may be divided by subheadings. It should provide a concise and precise description of the experimental results, their interpretation, as well as the experimental conclusions that can be drawn.

## 11. Conclusions

This literature review provides needed background information on wheelchair transportation safety for a new audience: people trying to design integrated wheelchair seating stations for AVs that can be used safely and independently by people who remain seated in their wheelchairs. Much of the early research in this area is still relevant today, partly because limited research has been conducted in the past decade. Reviewing how wheelchair safety and accommodation have been addressed in current vehicles is the starting point for developing innovative solutions that can be used in AVs. This section is mandatory.

## Figures and Tables

**Table 1:** Assessment of WTORS by independent use, crashworthiness level, and wheelchair/vehicle compatibility.

WTORS Type	Independent Use	High g and Low g Crashes?	Any Combination of Wheelchair and Vehicle?
4-pt strap tiedown paired with seatbelt	No	Yes	Yes
Docking station paired with seatbelt	Yes	Yes	No
Rear-facing stations	Yes	No	Yes
UDIG docking paired with automatic seatbelt	Yes	Yes	Yes

**Table 2:** Summary of previous modeling work related to wheelchair occupant protection.

Model	U Virginia	U Pittsburgh	U Louisville	UMTRI
References	[31]	[34]	[32,33,35,36,37]	[15,39]
Figure	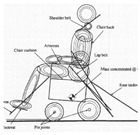	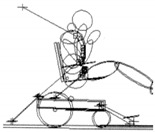	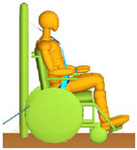	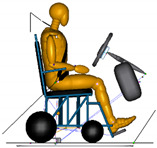
Software	DYNAMAN	DYNAMAN	MADYMO	MADYMO
Wheelchair	Surrogate wheelchair	Commercial Power WC	Commercial Manual Wheelchair	Surrogate wheelchair
Occupant	H-III 50th Male	H-III 50th Male	H-III 50th Male, 5th Female, 95th Male	H-III 50th Male, 5th Female
Restraint	3-point belt	3-point belt	3-point belt	3-point belt, driver airbag
Impact type	Frontal	Frontal	Frontal, rear	Frontal
Applications	Tiedown stiffness, tiedown position, wheelchair stiffness, sled pulse	Surface stiffness, seatback angle	Belt angle	Unbelted and belt misuse, airbag effect, oblique impact

**Table 3:** Occupied height for different types of wheeled mobility devices (adapted from [42]).

Type	Sample	Mean (SC)	Min	5%ile	10%ile	Median	90%ile	95%ile	Max
Manual	276	1249 (77)	1029	1123	1144	1253	1347	1376	1459
Power	189	1274 (81)	1000	1140	1153	1281	13734	1392	1492
Scooter	30	1321 (71)	1218	1220	1242	131	1434	1477	1513
All	495	1263 (80)	1000	1130	1152	1267	1360	1385	1513
